# Corneal Stromal Regeneration: A Review of Human Clinical Studies in Keratoconus Treatment

**DOI:** 10.3389/fmed.2021.650724

**Published:** 2021-02-23

**Authors:** Mona El Zarif, Jorge L. Alió, Jorge L. Alió del Barrio, Maria P. De Miguel, Karim Abdul Jawad, Nehman Makdissy

**Affiliations:** ^1^Optica General, Saida, Lebanon; ^2^Division of Ophthalmology, Universidad Miguel Hernández, Alicante, Spain; ^3^Faculty of Sciences, GSBT Genomic Surveillance and Biotherapy Team, Mont Michel Campus, Lebanese University, Beirut, Lebanon; ^4^Doctoral School of Sciences and Technology, Lebanese University, Hadath, Lebanon; ^5^Cornea, Cataract and Refractive Surgery Unit, Vissum (Miranza Group), Alicante, Spain; ^6^Cell Engineering Laboratory, IdiPAZ, La Paz Hospital Research Institute, Madrid, Spain

**Keywords:** stem cells, regenerative medicine, corneal bioengineering, corneal stem cell therapy, keratoconus, autologous adipose-derived adult stem cells, cross-linked collagen, corneal surgery

## Abstract

The use of advanced therapies with stem cells to reconstruct the complex tissue of corneal stroma has gained interest in recent years. Besides, collagen-based scaffolds bioengineering has been offered as another alternative over the last decade. The outcomes of the first clinical experience with stem cells therapy on corneal stroma regeneration in patients with advanced keratoconus were recently reported. Patients were distributed into three experimental groups: Group 1 (G-1) patients underwent implantation of autologous adipose-derived adult stem cells (**ADASCs)** alone, Group 2 (G-2) received a 120 μm decellularized donor corneal stromal laminas, and Group 3 (G-3) received a 120 μm recellularized donor laminas with **ADASCs**. A follow up of 36 months of clinical data, and 12 months of confocal microscopy study was performed, the authors found significant clinical improvement in almost all studied mean values of primary and secondary outcomes. Corneal confocal microscopy demonstrated an increase in cell density in the host stroma, as well as in the implanted tissue. Using different approaches, allogenic small incision lenticule extraction **(SMILE)** implantation was applied in cases with advanced keratoconus. Some authors reported the implantation of **SMILE** intrastromal lenticules combined with accelerated collagen cross-linking. Others performed intrastromal implantation of negative meniscus-shaped corneal stroma lenticules. Others have compared the outcomes of penetrating keratoplasty (**PKP**) vs. small-incision Intralase femtosecond (IFS) intracorneal concave lenticule implantation (**SFII**). Femtosecond laser-assisted small incision sutureless intrasotromal lamellar keratoplasty (**SILK)** has been also investigated. The published evidence shows that the implantation of autologous **ADASCs**, decellularized or recellularized human corneal stroma, allogenic **SMILE** lenticules corneal inlay, and recombinant cross-linked collagen have shown initially to be potentially effective for the treatment of advanced keratoconus. In light of the present evidence available, it can be said that the era of corneal stromal regeneration therapy has been already started.

## Introduction

Cellular therapy and tissue engineering of the corneal stroma has gained interest over the last decade as a potential alternative treatment for corneal stroma diseases, such as corneal scarring, dystrophies, and ectasias, such as keratoconus (1).

However, the highly complex structure of the corneal stroma, which involves very specific considerations concerning transparency, biomechanics, and optical behavior related to its very particular anatomical features (2), limits the usefulness of many of these corneal substitutes generated in preclinical experimental studies in the real clinical practice (3). On the other hand, published evidence has already demonstrated in pioneer human clinical studies that autologous stem cells from extraocular sources are capable not only to survive and differentiate *in vivo* into adult human keratocytes but also to produce new collagen within the host stroma (1). Mesenchymal stem cells (**MSCs**) may improve preexisting scars or defects in corneal transparency as it has been demonstrated in animal models (4, 5). Such **MSCs** have also shown immunomodulatory properties in syngeneic, allogeneic, and even xenogeneic scenarios, findings that make even more attractive their use in corneal stromal regeneration experiences (6, 7). Additionally, acellular corneal extracellular matrix (**ECM**) has been demonstrated to be an excellent scaffold for mesenchymal cells, acting as a carrier when implanted into the human cornea (8).

For all these reasons, cellular therapy of the cornea is to be considered as a promising advanced therapeutic approach for corneal diseases. However, the use of autologous human ocular keratocytes has many disadvantages, such as difficulties in its isolation, limitations in the availability of cells in high populations, and inefficient cell subculture (9). Corneal stromal stem cells have the same limitation of requiring healthy corneal donor tissue. Meanwhile, stem cells from extraocular sources have demonstrated that they may be a more favorable and adequate source for the regeneration of the human corneal stroma (1, 4, 5, 10–14).

This review aims to summarize the present status of the human clinical studies reported in the peer-reviewed literature on the topic of human corneal stromal regeneration, corneal enhancement therapy, and the immediate trends that such studies have opened new perspectives in the therapy of corneal stromal diseases, particularly in keratoconus.

## Background

The first human clinical trials using an extraocular source of **MSCs** for corneal stem cell therapy in advanced keratoconus cases were performed and reported recently by our group (1, 10–14), based on previous successful animal studies performed in part by the same research group (4, 5). In such preclinical studies, it has been shown that adult **MSCs** from human adipose tissue is an ideal source since they satisfy many requirements, such as easy access, high cell retrieval efficiency, and high differentiation capacity. They are known as “Human Adipose-Derived Adult Stem Cells” (**h-ADASCs**), these cells can be differentiated into different cell types, such as keratinocytes, osteoblasts, chondroblasts, myoblasts, hepatocytes, neurons, among others (4, 9).

Also, these cells have shown immunomodulatory properties in autologous, allogenic, and xenogeneic scenarios (6, 7). More than a decade ago, we found that **h-ADASCs** transplanted into damaged rabbit corneas were able to differentiate into corneal keratocytes and produced collagen and keratocan which are characteristics of the corneal stroma (4). To provide such collagen in an adequate volume to restore corneal thickness, protocols have been used to obtain corneal decellularized matrices as they provide a more natural environment for the growth and differentiation of **MSCs** compared to synthetic scaffolds (8, 15, 16). The effectiveness in this regard of sodium dodecyl sulfate decellularization on the human donor cornea has also been demonstrated in previous studies (8, 10, 11, 16). Such findings demonstrated that human cornea scaffolds provide a more adequate and natural environment for the growth and differentiation of cells, compared to synthetic scaffolds (8, 16). The use of **h-ADASCs** to repopulate scaffolds was also attempted (4, 5, 8, 16). We were able to demonstrate that the implanted cells survive at least 12 weeks after transplantation and they also differentiate into human keratocytes in the experimental animal model (16).

These experimental studies opened the translational of this concept from the animal into the human as a new therapy for human corneal disease, using the advanced keratoconus disease as the model for this type of advanced therapy.

## Cellular Therapy of the Corneal Stroma in Keratoconus

**MSCs** obtained from a simple procedure of liposuction followed by the isolation, characterization, and culture of autologous adipose tissue stem cells (4, 17, 18) were used. Corneal laminas obtained from human corneas IntraLase femtosecond (**IFS)** not suitable for corneal transplantation, duly decellularized and recellularized following an adequate corneal lamina protocol were also used (10, 11, 13, 14, 16, 19). The implants were performed into a mid-corneal stroma pocket created also with a femtosecond laser in corneas with advanced keratoconus. Patients were implanted with either adipose-derived adult stem cells (**ADASCs)** alone or with decellularized or recellularized laminas. The outcome at 1 month in all cases was an optically transparent autologous stromal graft, remaining transparent at 1 and 3 years (11, 14). The use of *in vivo* corneal confocal microscopy was very useful throughout the experience to observe the evolution of implanted **ADASCs** as well as the evolution of implanted tissue (13).

### Intrastromal Implantation of Autologous ADASCs Alone

#### Autologous ADASCs Isolation, Characterization, and Culture

The group of Jorge Alió performed a clinical trial phase 1 in five patients with advanced keratoconus. Standard liposuction under local anesthesia was performed and ~250 ml of adipose tissue was obtained from each patient. Processing of adipose tissue was performed according to the methods described in previous reports (1, 10–14, 17, 18). From 60 to 80 h before transplantation, quiescence of the **ADASCs** was induced by reducing the amount of serum in the culture media to 0, 5% to transplant them in a physiological state closer to non-proliferative natural stromal keratocytes. Quiescence and the absence of apoptosis and aneuploidy as well as blocking the pro-inflammatory and inducing the anti-inflammatory **ADASCs** properties, and the absence of infection as quality controls were verified as the authors described in the previous article. 3 × 10^6^ cells per patient were prepared in phosphate-buffered saline (**PBS**), the cell dose was based on the evidence observed in previous studies (1, 10–16), and the cells were transplanted directly *in situ*.

#### Surgical Procedure

**Figure 1:**
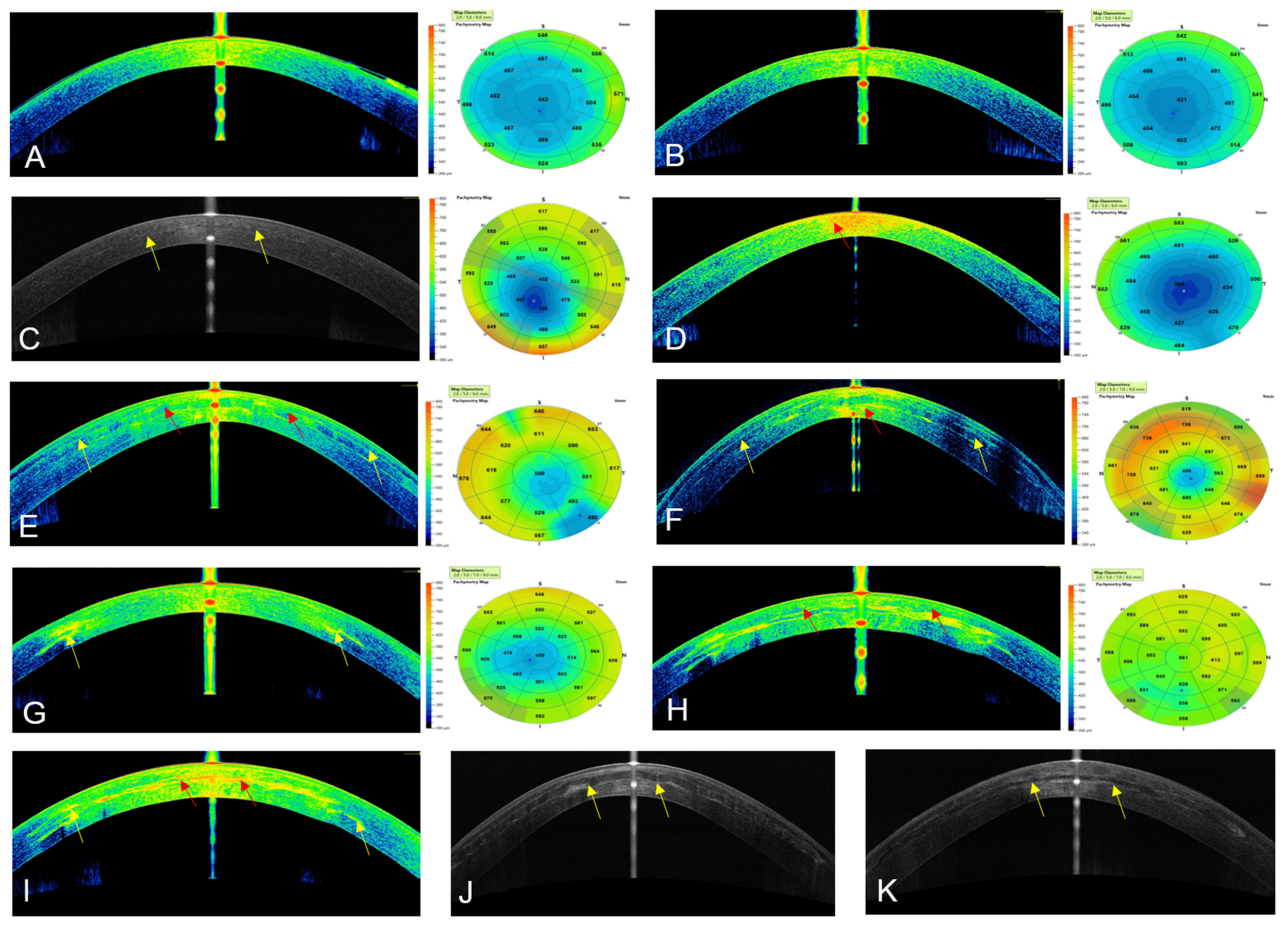
Cornea Visante optical coherence tomography images and pachymetric maps. **(A)** Preoperative **OCT** in case 1, G-1. **(B) OCT** at 12 months post-op of the same patient, notice the transparency of the cornea. **(C) OCT** at 36 months post-op with case 2, G-1. Observe the transparency of the cornea, the (yellow arrows) demonstrate the limit of the flap. **(D)** Preoperative **OCT** in case 7, G-2, Notice the highly reflective area in the corneal stroma (red arrow). **(E,F) OCT** at 12 months and 36 postoperative in case 7. The improvement of the reflective band of neo-collagen in the periphery of the implanted lamina (yellow arrows) can be seen. The reflectance of the neo-collagen band (red arrow) is higher at 12 months in the center of the implanted lamina. **(G)** Preoperative **OCT** in case 13, G-3. The reflective paracentral scars can be noticed (yellow arrow). **(H,I) OCT** at 12 and 36 months post-op. The high reflectance of the neo-collagen band (red arrow) can be seen more evident at 36 months with this patient in the center of the lamina. Notice the improvement of the preoperative paracentral scars at 36 months post-op (yellow arrows). **(J) OCT** in Case9, G-2, and **(K)** in Case 12, G-3 at 36 months post-op. (yellow arrows) shows the limits of the laminas.

**Figure 2:**
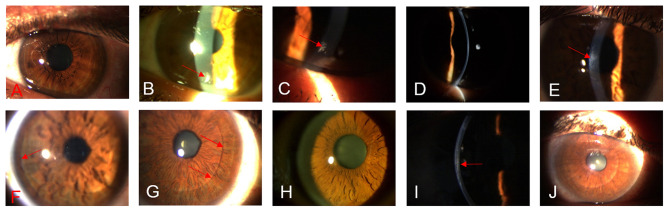
Biomicroscopic changes between the preoperative up to 36 months post-operative. **(A)** G-1, case 4, OS, shows the transparency of the cornea in the preoperative. **(B)** G-1, case 4, shows the transparency of the cornea. The presence of a peripheral surgical scar (red arrow) at 1month post-op can be noticed. **(C)** G-1, case 4, shows the stability of the peripheral scaring (red arrow) in the cornea at 12 months post-op. **(D)** G-1, case 4, shows the transparency of the cornea at 36 months post-op. **(E)** G-2, case 9, OD, shows the presence of a slight paracentral scar( red arrows) at the pre-operative. **(F)** G-2, case 9, 12 months post-op, observe the improvement in the transparency of the implanted lamina, (red arrow) represent the periphery of the lamina. **(G)** G-2, case 9, 36 months post-op, notice the marked improvement in the transparency of the lamina, the periphery of the sheet becomes not very marked (red arrow). **(H)** G-3, case 12, OD, shows the transparency of the cornea in the preoperative. **(I)** G-3, case 12, 12 months post-op. The (red arrow) indicates the implanted recellularized lamina. **(J)** G-3, case 12, 36 months post-op. The (red arrow) indicates the periphery of the recellularized lamina. Notice the improvement of the transparency of the implanted tissue.

Topical anesthesia was administered. **IFS** femtosecond laser (AMO Inc, Irvine, CA) 60 Khz. IntraLase was used in a single-pass mode for the recipient corneal lamellar dissection. At the medium depth of the thinnest preoperative pachymetry point measured by an anterior segment optical coherence tomography (**AS-OCT)** (Visante, Carl Zeiss, Germany), an intrastromal laminar cut of 9.5 mm of diameter was created and dissected. An injection of 3 million autologous **ADASCs** in 1 ml **PBS** in the stroma pocket using a 25-G cannula was performed. No corneal sutures were used (Figures 1A–C, 2A–D) (1).

#### Clinical Results

**Figure 3:**
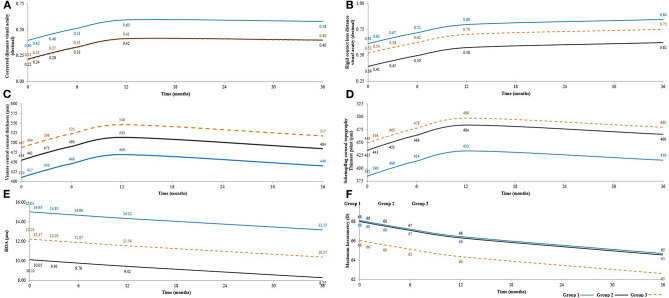
Shows statistical mean results after 3 years follow-up in G-1, G-2, and G-3, respectively **(A)** Shows an amelioration in **CDVA** (corrected distance visual acuity) (decimal), mean values in the pre-operatives were (0.40, 0.22, 0.21), at 12 months post-operatives mean values were (0.60, 0.42, 041) and (0.58, 0.40, 0.40) at 36 months post-operative. Desviation Standard = 0.151. **(B)** Shows an upgraded in **CLDVA** (decimal equivalent to logMar scale) from the pre-operative (0.61, 0.39, 0.52) until reach a maximum mean values at 36 months post-operative (0.84, 0.62, 0.75). Desviation Standard = 0.180. **(C)** Presents Visante **CCT** (central corneal thickness) (μm) mean values in the pre-operative (410, 454, 487) (μm), at 12 months post-operative, these values reached a maximum average (469, 513, 546) (μm), then at 36 months post-operative, they were established with the following average values (440, 484, 517) (μm). Desviation Standard = 62.940. **(D)** Presents the Scheimpflug corneal topography thinnest point (μm) mean values in the preop (385, 435, 449) (μm), there were an increase in the topographic mean values till 12 months post-operative (433, 484, 498) (μm), than at 36 months post-operative, a small decrease was obtained with this mean values (416, 466, 480) (μm). Desviation Standard = 67.966. **(E)** Observe the amelioration in RMS HOA (high order aberration in μm) in mean values at the pre-operative values (15.01, 10.11, 12.23), at 36 months post-operative (13.15, 8.25, 10.37). Desviation Standard = 4.530. **(F)** Observe **Kmax** , the pre-operative mean values (68, 68, 66), there was a mean values of 2 diopters more flatter at 12 months post-operative (66, 66, 64) regarding the preoperative, followed by another mean value of 1 diopter of flattening till 36 months post-operative (65, 65, 63). Desviation Standard = 8.250.

No complications were observed during the 3 year follow-up. No adverse events, such as haze or infection were encountered. Full corneal transparency was recovered within the first postoperative day in all patients (Figures 2A–D). New collagen production was observed as patchy hyperreflective areas at the level of the stromal pocket (1, 11). No patient lost lines of visual acuity. All cases presented an improvement in their Unaided distance visual acuity (**UDVA**) of (0.08, 0.14, 0.12), also in their corrected distance visual acuity (**CDVA**) of (0.11, 0.2, 0.18) (Figure 3A), and rigid contact lens distance visual acuity (**CLDVA**) of (0.11, 0.19, 0.23) (Figure 3B), in decimal lines mean value equivalents in LogMar scale at 6, 12, and 36 months of follow up (14).

**Figure 4:**
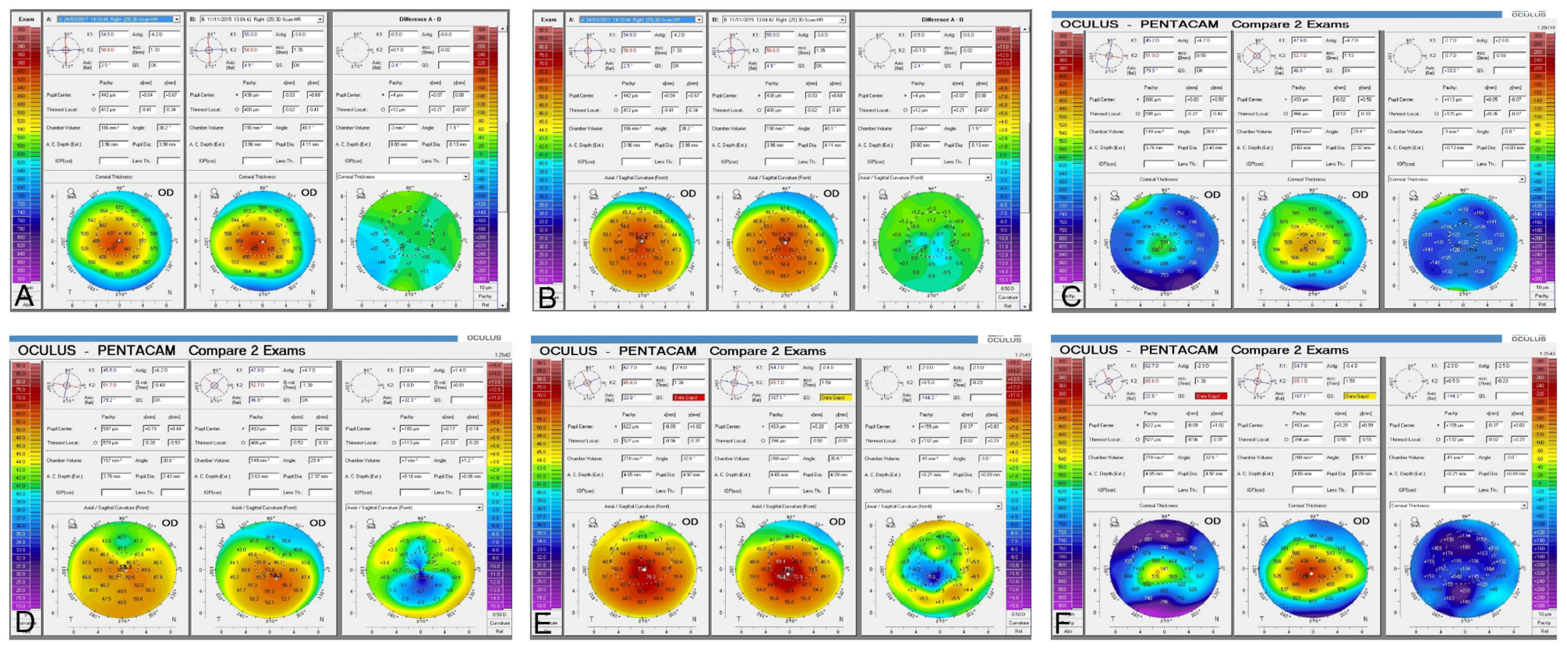
Corneal topography (Pentacam) comparison between preoperative, 12 and 36 months post-op. **(A)** Comparative paquimetric examinations (right side) among the preoperative (middle) and 12 months post-op (left side) in case 1, G-1. Observe the modest improvement of the corneal thickness **(B)** Preoperative (middle) and 12 months post-op (left side) sagittal curvature comparative exams (right side), in case 1, G-1. Notice the modest improvement in the keratometric parameters of the cornea. **(C)** Comparative paquimetric examinations (right side) among the preoperative (middle) and 12 months post-op (left side) in case 6, G-2. Observe the improvement of the corneal thickness. **(D)** Preoperative (middle) and 36 months post-op (left side) sagittal curvature comparative exam (right side), in case 6, G-2. Notice the improvement in the keratometric parameters of the cornea. **(E)** Sagittal curvature comparative exam (right side) among the preoperative (middle) and almost 3 years post-op (left side) in case 12, G-3. Enhancement of the keratometric parameters can be observed. **(F)** Comparison pachymetric exam (right side) among the preoperative (middle) and 3 years post-op (left side) in case 12, G-3. The markable enhancement of the paquimetric parameters can be noticed.

Results of Central corneal thickness (**CCT**) (μm) was measured by **AS-OCT** (Visante, Carl Zeiss) (Figures 1, 3C), Scheimpflug corneal topography Thinnest point (**Thinnest point**) (μm) (Figures 3D, 4A) and Cornea Volume **(CV**) (mm^3^) showed an increase in mean values in all patients of this group (36, 59, 30) (μm), (29, 48, 31) (μm), and (3, 3, 4) (mm^3^) during the 6, 12, and 36 months of follow-up (14).

On the other hand, the Refractive sphere (**Rx Sphr**) (D) presented an improvement of (0.8, 1.3, 1.1) myopic diopters at 6, 12, and 36 months. Meanwhile, the refractive cylinder (**Rx Cyl**) (D) remained almost stable 12 months post-operative followed by a deterioration of 0.5 (D) until 36 months post-operative regarding the pre-operative mean values (14).

Also, an improvement in mean values was observed during the 3 year follow up in third-order aberration RMS **(3rd order RMS)** (μm), fourth-order aberration RMS **(4th order RMS)** (μm), high order aberration RMS **(HOA RMS)** (μm) (Figure 3E) and Low order aberration RMS **(LOA RMS)** (14).

Additionally, results of Anterior mean keratometry (**Anterior Km**) (D) (Figure 4B) presented a modest improvement of mean values of 1 (D) to 12 months post-operative and another 1 (D) at 36 months post-operative regarding the pre-operative mean values. The authors detected stability in mean values of Posterior mean keratometry (**Posterior Km**) (D) up to 36 months post-operative. Nevertheless, they found a flattening in mean values of 2 (D) in maximum keratometry (**Kmax**) (D) (Figure 3F) up to 12 months post-operative, followed by 1 (D) up to 36 months post-operative regarding the pre-operative mean values. Finally, a deterioration of −0.3 (D) was obtain**ed** at 12 months post-operative in the topographic cylinder **(Topo Cyl)** (D), at 36 months post-operative they found the same pre-operative mean values (14).

### Collagen-Based Scaffolds

#### Decellularization and Recellularization of Human Corneal Stroma Laminas

In this phase 1 of our clinical trial in advanced keratoconus, five patients received decellularized human corneal stroma laminas only, and four patients received human corneal stroma recellularized with autologous **ADASCs**. Laminas were obtained from donor corneas from an eye bank. The epithelium was removed mechanically, the anterior corneal stroma was cut by 60 kHz IntraLase **IFS** femtosecond laser (AMO, Santa Ana, CA), 2–3 consecutive laminas 120 μm thick and 9.0 mm in diameter were obtained, a surface that contains the Bowman membrane and a deeper one without this layer. The decellularization protocol was based on previous publications (8, 10, 20). The laminas for patients who received recellularized tissues [(1, 10–14), del Barrio et al., 2015], were placed in tissue culture medium for recellularization 24 h before implantation with autologous **ADASCs** (0.5 × 10^6^ cells were cultured on each side of the laminas). After finishing the recellularization process, the laminas were washed with **PBS** at room temperature before implantation (10).

#### Surgical Procedure: Lenticule Implantation

Topical anesthesia was applied with oral sedation for all surgeries. The 60-kHz IntraLase **IFS** femtosecond laser was used in single-pass mode. Assisted corneal dissection was done with a 50° anterior cut, the lamina was inserted through the corneal intrastromal pocket, then centered and unfolded through gentle tapping and massaging from the epithelial surface of the cornea. In those cases that received a recellularized lamina, the pocket was irrigated immediately before and after insertion with a solution containing an additional 1 million autologous **ADASCs** in 1 ml of **PBS** with a 25G cannula. One interrupted 10/0 nylon suture was performed and was then removed 1 week after the operation (Figures 1D–K, 2E–J) (10).

#### Clinical Results

The authors did not observe any complications during the 3 year follow-up, with the exception that the implanted lamina showed a mild early haziness during the first postoperative month. This issue was related to mild lenticular edema. Corneal recovery and full transparency were observed within the third post-operative month in all patients. No adverse events of any type were observed over the 3 years (Figures 2E–J) (14).

All patients with decellularized or recellularized laminas gained an improvement during 6, 12, and 36 months of follow up, the enhancement in **UDVA** was (0.08, 0.13, 0.11) in decimal values nearly equivalent to one line in logMar scale with decellularized and recellularized laminas, **CDVA** was (0.11, 0.2, 0.18) with decellularized lamina and (0.12, 0.2, 0.19) with recellularized ones, equivalent to 1–2 lines in LogMar scale (Figure 3A), as well **CLDVA** means improvement was (0.11, 0.19, 0.23) with decellularized laminas and (0.1, 0.18, 0,23) with recellularized laminas (Figure 3B), equivalent to 1-2 lines in LogMar scale. Differences among the groups were summarized in Table 1.

**Table 1:** Shows the difference in mean values among (G-1)–(G-2), (G-1)–(G-3), and (G-2)–(G-3) of the variables of the study.

	**(G-1)–(G-2)**	**(G-1)–(G-3)**	**(G-2)–(G-3)**
UDVA (LgogMar)	0.07	0.07	0.00
CDVA (LgogMar)	0.18^*^	0.19^*^	0.01
CLDVA (LgogMar)	0.22^*^	0.10	−0.12^*^
3rd order RMS	4.65^*^	3.54^*^	−1.11
4th order RMS	1.28	−0.17	−1.45^*^
HOA RMS	4.9	2.78	−2.12
LOA RMS	−3.87^*^	1.32^*^	5.19
Visante CCT (μm)	−44.00^*^	−77.00^*^	−33.00
Thinnest point (μm)	−51.00^*^	−65.00^*^	−14.00
CV (mm^3^)	−5.00^*^	−5.00^*^	0.00
Rx Sphr (D)	0.10	−0.10	−0.20
Rx Cyl (D)	−1.00^*^	−0.60^*^	0.40
Kmax (D)	0.00	2.00	2.00
Topo Cyl (D)	1.1	0.9	−0.2
Anterior Km (D)	−3.00	1.00	4.00
Posterior Km (D)	1.3^*^	0.2	−1.1^*^

* *Indicates a statistically significant difference between the compared groups*.

Results of **CCT** (μm) (Figure 3C)**, Thinnest point** (μm) (Figure 3D) showed an improvement of (36, 59, 30) (μm), and (29, 49, 31) (μm), respectively, also, mean value results **of CV** (mm^3^) demonstrated an improvement of (2, 2, 3) (mm^3^) in both laminas' groups at 6, 12, and 36 months of follow up (14). The results were statistically significantly better in G-2 and G-3 comparing with G-1 (Table 1).

**Figure 5:**
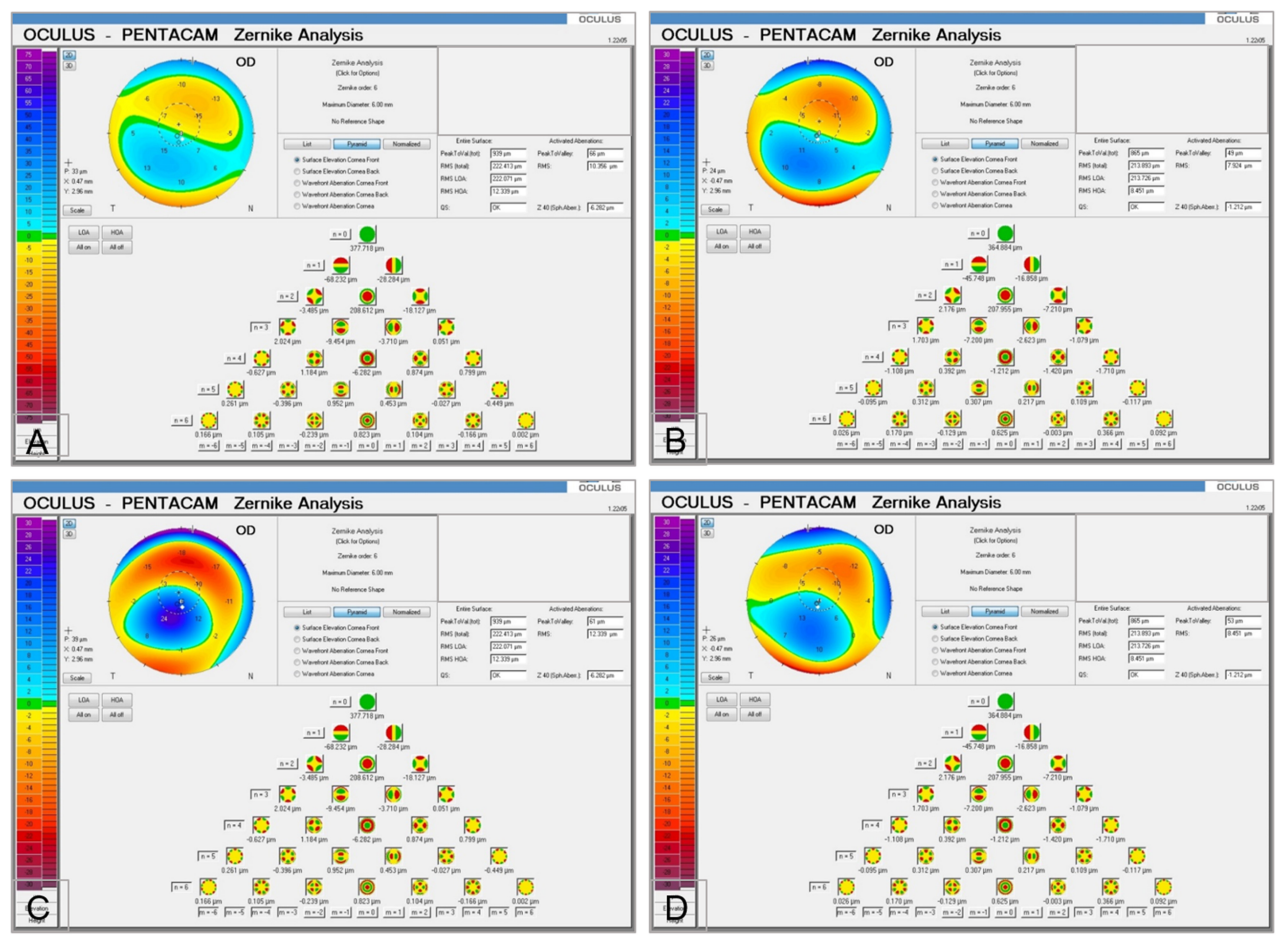
Corneal Aberrometry with case 5, G-2. **(A,B)** (Pentacam) Third-order aberration RMS **3rd order RMS** (μm). Observe the improvement in mean values from the preoperative (10.356) (μm) till (7.924) (μm) at 36 months post-op. **(C,D)** (Pentacam) high order aberration RMS **HOA RMS** (μm). Notice the amelioration between the preoperative mean values (12.339) (μm) and 36 months post-op (8.451) (μm).

Besides the **Rx Sphr** (D), **Rx Cyl** (D), Results of **3rd order RMS** (μm) (Figures 5A,B), **4th order RMS** (μm), **HOA RMS** (μm) (Figures 3E, 5C,D), and **LOA RMS** (μm). **Anterior Km** (D), **Posterior Km** (D), **Kmax** (D) (Figure 3F), and **Topo Cyl** (D) with decellularized and recellularized laminas showed results close to patients' results with implantation of **ADASCs** (14) (Table 1).

More information about the comparative results among the three groups was summarized in Table 1.

### Confocal Microscopy Study

#### Methodology

**Figure 6:**
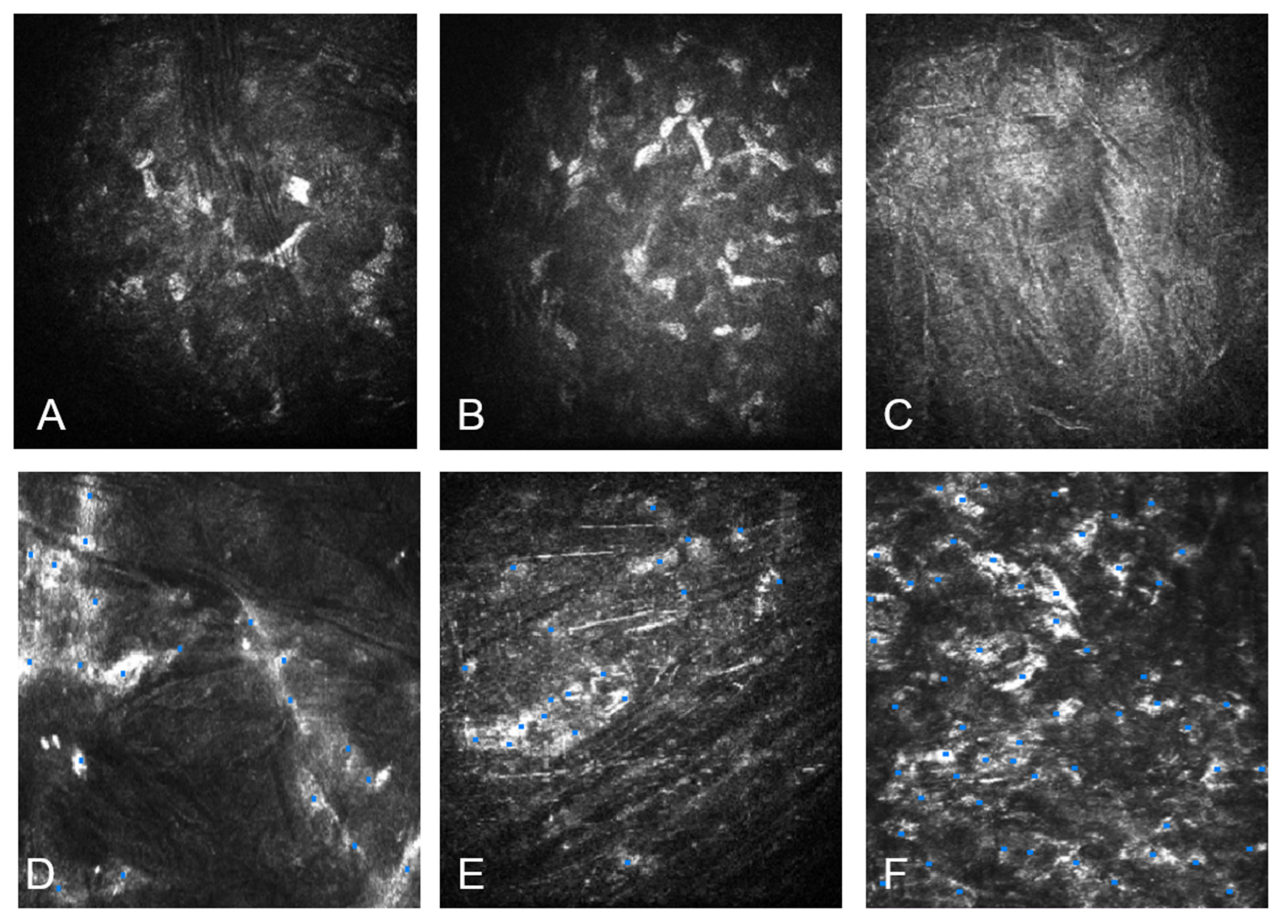
Confocal microscopy study with HRT3 RCM (Heidelberg). **(A)** Confocal microscopy of the anterior corneal stroma with case 1, G-1. Notice the few nuclear corneal cell density. **(B)** Nuclear cell density in the anterior stroma with case 1 at 12 months post-operative. Notice the increment of the cell density. **(C)** Acellular implanted lamina with case 5, G-2 at 1 month post-operative. **(D)** Recellularization of the anterior surface of the implanted decellularized lamina in the same case 5 at 12 months postoperative. **(E)** The posterior surface of the recellularized lamina with case 13, G-3 at 1 month after the operation: the presence of few **ADASCs** with similar morphology of keratocytes. **(F)** The posterior surface of the recellularized lamina with case 13 at 12 months after surgery: Observe the abundant number of stromal cells.

***The confocal microscope HRT3*** RCM (Heidelberg) with Rostock Cornea Module was used. An area is known as the “region of interest” was determined (13, 21). The same area was taken in all the studied pictures (0.1 mm^2^). Nuclei cell count was performed with 50% brightness and contrast, and the nucleus of cells that were more illuminated, more refringent was selected (13, 22, 23), the selected nucleus had well-defined edges (Figures 6A,B) (13, 24).

##### ADASCs Counting

The counting method for transplanted **ADASCs** was performed similarly 1 month after the surgery. **ADASCs** appeared rounded in shape, more voluminous, and refringent (13).

##### Cell Count on Decellularized and Recellularized Laminas

**O**ne month after surgery, the decellularized laminas appeared without well-defined cell structures, then they were considered acellular. With the recellularized ones, the presence of a few **ADASCs** was observed, and they had a similar morphology to normal keratocytes nucleus and these structures were counted as cell nuclei (Figures 6C–F) (13).

#### Results

##### Autologous ADASCs Implantation

Morphological results demonstrated that **ADASCs** appeared rounded in shape, more voluminous, and refringent than the host corneal keratocytes up to 6 months post-operative. Also, statistical results at 12 months after the operation showed a gradual statistically significant increase in the cellular density at the anterior, mid, and posterior host stroma when comparing the preoperative values (Figures 6A,B) (13). Moreover, the authors detected the presence of a small fibrotic tissue at 3 months postoperative in 2 out of the 4 patients, but a full recovery of the corneal stroma was observed at 12 months follow-up (13).

##### Decellularized and Recellularized Corneal Laminas Implantation

Decellularized laminas appeared acellular during the first months unlike the recellularized ones, which showed similar structures to corneal keratocytes, where the number of cells increased during the 12 months follow-up. The anterior, mid, and posterior surfaces of the decellularized and recellularized laminas became more colonized by keratocyte-type cells until they showed similar morphology of normal corneal keratocytes (Figures 6C–F) and reached statistically significant increased cell density values in comparison to the first postoperative month. 12 months after surgery, the cell density of the anterior, and posterior corneal stroma showed a statistically significant increase in the number of keratocytes close to that found in a normal cornea (13). The confocal microscopy study also showed the formation of fibrotic tissue in non-central corneal areas in almost all of the cases implanted with decellularized lamina's and recellularized ones, but no significant association was found between recellularization and cell density and the presence of such fibrotic tissue (13).

## Allogenic Smile Lenticule Corneal Inlay Implantation

The implantation of these stromal lenticules has been proposed as a way to reverse the effect of previous laser refractive surgery (25, 26), to treat different types of ametropia (27), and also as a therapy for cornea ectatic disorders such as keratoconus (28–33).

The hyperopic algorithm for small incision lenticule extraction **(SMILE)** produces negative meniscus lenticules thicker from the center toward the periphery, the implantation of these negative meniscus shaped lenticules in an *ex vivo* study on human corneas caused a reproducible flattening of the central cornea and increase of the stromal thickness, both desirable effects of any procedure for treating keratoconus eyes (28–33). The myopic correction algorithm for **SMILE** produces positive meniscus lenticules, thicker in the center and gradually becoming thinner toward the periphery. Conversely, positive meniscus lenticules have been used to correct hyperopia in humans (28–33).

### Lenticular Implantation Combined With Collagen Cross-Linking

#### Methodology

Ganesh et al. in one pilot clinical study were reported including 6 patients with advanced keratoconus, to evaluate the outcomes of femtosecond intrastromal lenticular implantation (**FILI**) from corneal stroma donor tissue, combined with accelerated collagen cross-linking (28, 29). Only lenticules with spherical myopic refractive errors were chosen for cryopreservation, the details of the methods have been described in previews publication (28, 29). The tissue was soaked in 0.25% riboflavin dye and kept aside and a 3-mm corneal trephine was used to punch the center of the lenticule to obtain a donut-shaped tissue. After that, topical anesthesia was applied. A pocket was created with Visumax femtosecond laser (Carl Zeiss Meditec AG), with 7.0–8.0 mm diameter at 100 μm depth, then the donut-shaped lenticule was inserted gently into the pocket through the 4 mm superior incision and finally, the eye was exposed to ultra-violet radiation (**UV**-A) using the Avedro Cross-linking (**CXL)** system. Follow-up of patients was performed for a mean period of 190 days.

#### Results

The authors did not observe any adverse reaction, such as haze, infection, or allogeneic graft rejection during the 3 years postoperative follow up. Clinical improvement was observed in all patients, no eye lost lines of corrected distance visual acuity, and amelioration was detected in **UDVA** from 1.06 to 0.38 (logMar scale), **CDVA** from 0.51 to 0.2 (logMar scale), and manifest spherical equivalent from −3.47 to −1.77 D. There was flattening of **Km** in 3- and 5-mm zones by 3.42 and 1.70 (D), respectively. Also mean pachymetry in the central and mid-peripheral zones increased by 18.3 and 33.0 (μm), respectively. All patients had improvement in high-order aberrations, especially in coma (29).

### Femtosecond Laser-Assisted Stromal Lenticule Addition Keratoplasty (SLAK)

#### Surgical Technique

Mastropasqua et al. (30, 31) reported the visual and refractive outcomes after the intrastromal implantation of negative meniscus-shaped corneal stroma lenticules (thicker in the periphery than in the center) for advanced keratoconus patients (*n* = 10) as an alternative approach to classical corneal transplantation techniques. They termed this technique as stromal lenticule addition keratoplasty (**SLAK**). These allogeneic lenticules (6.7 mm in diameter) were dissected with a Femtosecond laser (Visumax, Zeiss, Germany) from donor corneas, and implanted into a Femto dissected pocket within the patient's recipient cornea.

#### Results

At 6 months, **UDVA** and **CDVA** showed statistically significant improvements from 1.58 ± 0.36 to 1.22 ± 0.37 and from 1.07 ± 0.17 to 0.70 ± 0.23 (logMAR), respectively. 8/10 eyes showed an improvement in **UDVA** that ranged between 1 and 3 lines. All except one eye presented improved **CDVA**. These allogeneic negative in refraction, meniscus-shaped lenticules induced a generalized flattening of the cone, with a reduction of **Anterior Km** from 58.69 ± 3.59 to 53.59 ± 3.50 (D) at 6 months after surgery. Postoperative **AS-OCT** showed a significant increase in the thickness of the central and mid-peripheral corneal mean **CCT** values increased from 406 ± 43 to 453 ± 39 (μm) at the end of the follow-up (30, 31). Confocal microscopy (HRT II Rostock Cornea Module, Heidelberg Engineering GmbH, Germany) showed identification of the lenticule–host interfaces in all cases. Both anterior and posterior lenticule interfaces were characterized by dishomogeneous hyperreflectivity and acellular aspect, this reflectivity decreased over time with all the cases. At 1 week an activated and lower reflective elongated keratocytes within the lenticule lamellae were observed. At 6 months, keratocyte morphology appeared similar to normal, keratocytes structures were observed within the implanted lenticules with regular shape and reflectivity, whereas the extracellular tissue presented normal transparency (30, 31).

### Intracorneal Concave Lenticule Implantation With PKP and SFII

#### Surgical Technique

Xingwu et al. realized a comparative study with a total of 31 consecutive patients (31 eyes) with progressive keratoconus, 11 patients underwent small-incision femtosecond laser-assisted intracorneal concave lenticule implantation **SFII** (SFII group), and 20 of them underwent penetrating keratoplasty (**PKP**) (PKP group).

SFII group: The donor cornea was placed into an artificial anterior chamber, and a lamellar incision was made with a femtosecond laser (32, 33). An anterior incision was made with a diameter of 7.5 (mm) and was located with a depth of 320 (μm), then a myopia correction of −4.00 diopters was executed (69.59 μm thickness) with an excimer laser (WaveLight GmbH, Erlangen, Germany). The lenticule was then separated from the stromal bed and used as a graft. Then, **SMILE** treatment with a myopic correction of −0.75 (D) (28 μm thickness) was effectuated in the recipient cornea with 160 (μm) cap thickness, 7.8 (mm) cap diameter, and 7.8 (mm) lamellar cornea diameter (32, 33). Finally, the graft was implanted into the stromal pocket.

PKP group: PKP was performed under general anesthesia, the donor corneas were cut using a punch trephine on a Troutman guide. All the grafts had diameters between (7.0–8.0 mm). All grafts were closed with a running 24-bite 10-0 nylon suture.

#### Results

At 3 months postoperative in the **SFII** group, all patients improved their **UDVA** and **CDVA**, then they remained stable, at 24 months 82% of the operated patients from this group improved 3 lines, 9% gained 2 lines, and other 9% gained 1 line in Snellen scale. In the **PKP** group, all patients did not improve their **UDVA** during the 24 months follow-up, nevertheless, they improved their **CDVA**, only 60% improved 3 lines on the Snellen scale (33). Minimum central keratometry and maximum central keratometry improved (−3.9 ± 0.95) (D) and (−4.65 ± 2.04) (D) respectively, but less than with **PKP** results (33). Changes of the anterior corneal depth were (−0.12 ± 0.09) (mm) with **SFII** group, and (−0.46 ± 0.29) (mm) with **PKP** at 24 months (33).

The intraocular pressure (**IOP**) with the group **PKP** increased during the first-month follow-up and it declined up to the third month. In the **SFII** group, the **IOP** maintained stability during the first month. An increased in the corneal thickness was better in the **SFII** group than in the **PKP** group at 3 months after the surgery. Confocal microscopy study revealed the presence of dendritic cells in the subepithelial region, also, dendritic and inflammatory cells could be observed among graft and the host cornea up to 1 month with the group **SFII**, and up to 3 months with the **PKP** group (32, 33).

### IFS Assisted Small Incision Sutureless Intrastromal Lamellar Keratoplasty (SILK)

#### Surgical Technique

Pradhan et al. (34), using a different approach, proposed femtosecond laser-assisted small incision sutureless intrastromal lamellar keratoplasty (**SILK**) as an alternative to corneal traditional anterior lamellar or full-thickness corneal transplantation in keratoconus with one single patient, and 1 year follow up was presented. Intrastromal addition of a corneal lenticule was preceded by intrastromal extraction of stroma from the recipient cornea. The donor cornea was prepared using the VisuMax deep anterior lamellar keratoplasty (**DALK**) software module, and 50 microns myopic ablation was performed using a MEL-80 excimer laser (Carl Zeiss Meditec), to obtain a negative meniscus in the anterior surface. The recipient intrastromal pocket was created using a combination of the **DALK** and laser *in situ* keratomileusis (**LASIK**) flap software modules. The donor stromal lenticule was then implanted into the recipient through 3-mm small incision.No sutures were applied.

#### Results

With the **SILK** technique, an improvement in visual parameters was obtained, the preoperative **UDVA** was counting fingers, and the **CDVA** was (20/80, Snellen), One year postoperatively, **UDVA** changed to (20/80), and with the manifest refraction **CDVA** improved to (20/40). As well a reduction of 7,34 (D) in **Kmax** was obtained and resulting from a considerable thicker lenticule of 332 (μm) in the central cornea (34).

## Recombinant Cross-Linked Collagen for Corneal Enhancement

### Surgical Technique

Recent studies (35, 36) performed a phase 1 clinical trial in 10 patients with vision loss because of keratoconus or scarring, 9 eyes with keratoconus, and 1 eye with a scar. The cohort comprised eight men and two women (age range: 18–75 years), at the time of surgery. They used recombinant human collagen III-based (13.7% wt/wt) (**RHCIII**), scaffolds cross-linked with 1-ethyl-3-(3-dimethylaminopropyl) carbodiimide (**EDC**) and N-hydroxysuccinimide (**NHS**) (**EDC-NHS**) and fabricated into substitutes with the dimensions of a human cornea and transplanted into the anterior stroma. They performed anterior lamellar keratoplasty (**ALK**) under general or local anesthesia. Patients received these biomimetic substitutes (6.25–6.75 mm diameter, 500 μm thick) anchored with three to four overlying 10–0 nylon sutures and covered with a bandage lens. Bandage lenses and sutures were removed 5 weeks after surgery (35, 36).

### Results

After 6–7 months, the corneal substitutes were well-integrated into all 10 recipients, without adverse effects or complications, such as neovascularization, inflammation, or rejection. Remodeling of the thick substitutes was reported, resulting in the restoration of a seamless host–graft interface and a corneal surface becoming progressively smoother over time. At 24 months, they showed stable biointegration and corneal reepithelialization, but localized implant thinning that varied from 211 to 568 microns in thickness after 2 years and fibrosis in several patients. Vision improved in 6 out the 10 patients. Four years after the grafting (37), patient **CDVA** was 20/54 on average and a gain of more than the 5 Snellen lines of vision on an eye chart. At four years, the biosynthetic corneas showed steeper surface curvatures and were more irregular than that of donor tissue showing increased astigmatism (38).

## Discussion

Our research group demonstrated for the first time the feasibility of regenerative surgeries of the corneal stroma for advanced keratoconus, using autologous **ADASCs** injected into the corneal stromal pocket in cases with advanced keratoconus, confirming also the appearance of new collagen in the injected areas, that could be useful for repairing the corneal dystrophies, scars and could also slightly increase the thickness of the cornea (1, 11, 14).

Also, we demonstrated for the first time that decellularized laminas of a human corneal stroma, colonized or not by autologous **ADASCs**, can be implanted for therapeutic purposes on a clinical basis (Figures 1E–K, 4C–F). After 3 years of follow-up, no patient showed inflammation, rejection, or any evidence of scarring or haze (Figures 1, 2) (10, 11, 13, 14). Furthermore, there was an improvement in all the visual parameters with 1-2 lines in LogMar (Figures 3A,B). The results of the G-1 showed an increase of 30 (μm), 31 (μm), and 3 (mm^3^)in **CCT**, **thinnest point** and **CV**, respectively, statistical differences were significantly better obtained at 36 months in patients with implanted laminas, the **CCT** mean difference among G-1/G-2 was 44 (μm), nevertheless, this mean difference was 77 (μm) when comparing G-1/G-3 (Figure 3C; Table 1). The mean difference in the **thinnest point** was 51 (μm) among G-1/G-2 and 65 (μm) among G-1/G-3 (Figure 3D; Table 1). Also mean differences in **CV** were 5 (mm^3^) when comparing G-1/G-2, and G-1/G-3 (Table 1) (14). There was also an improvement in all the corneal aberrations (Figures 3E, 5), and corneal topographic parameters (Figure 4) (14).

The 12 months follow up of the confocal microscopy study showed the evolution of **ADASCs** nuclei and their morphological changes from being rounded shape and highly refringent cells at 6 months to fusiform structures and less nucleus refringent. These findings demonstrate in the human clinical model that the **ADASCs** implanted in the corneal human pocket have survived and have been able to differentiate into keratocytes (Figures 6A,B) (13). One year after the surgery, a gradual and significant increase (*P* < 0.001) was observed in the cellularity in the anterior, mid, and posterior stroma in G-1, as well in the anterior, and posterior stroma, and among the implanted decellularized/recellularized laminas in G-2 and G-3, in comparison with the preoperative density level (13). Such findings confirm the previous animal studies in which the post-mortem analysis demonstrated the presence and survival of these human cells and the human collagen produced by them in the rabbit cornea (4, 15).

Allogenic refractive lenticule corneal inlay using a **SMILE** lenticule combined or not with accelerated collagen cross-linking (28, 29, 31, 33), is a promising surgery, no inflammation, complication, opacification, vascularization, or infection were recorded. Another group performed a recent prospective study in Tehran (39), they reported a novel method for the treatment of advanced ectasia, with customized SMILE lenticule implantation with 22 cases of advanced keratoconus (shape of the lenticule was compound form or necklace). Corneal thickness showed a mean enhancement of 100.4 μm at the thinnest point measured by **AS-OCT**. An improvement in visual parameters was obtained, best **CDVA** improved from 0.70 (range 0.4–1) to 0.49 (range 0.3–0.7) at 6 months with all the patients, which was in coherence with the decrease in corneal aberration, also keratometry decreased from 54.68 ± 2.77 to 51.95 ± 2.21 (D) was obtained.

Stromal addition of **SMILE** created lenticules have been proven *ex vivo* and *in vivo* its feasibility, safety, and efficacy in the treatment of thinning disorders such as keratoconus (30, 31, 34). **SILK** induced in **Kmax** a flattening of 7,34 (D) and using negative meniscus-shaped lenticule addition induced also some flattening of the cone, the improvement was (5.1 D at 6 months) in **Anterior Km** better than the ones observed with Ganesh et al. (29), and planar than the lenticules implanted by El Zarif et al. (14).

The comparatives surgical procedures **SFII** and **PKP** resulted in a stable corneal volume, and improved visual acuity during the 24 month study period. **SFII** was less invasive and more efficient when compared with **PKP**. Using concave lenticule implantation improved the maximum central keratometry of (−4.65 ± 2.04) (D), this outcome was more planner than the laminas implanted with El Zarif et al. (14) and Jin et al. (33).

However, corneal thickness increase using planner lenticules (1, 10, 11, 14) is higher than the one using allogenic **SMILE** lenticule corneal inlay implantation combined or not with collagen Cross-Linking (29, 31, 33), besides, negative meniscus-shaped lenticules or donut-shaped lenticules are only available for pure central cones, as for eccentric cones (the ones are commonly seen in keratoconus) they would generate non-acceptable aberrations within the visual axis, unlike the planner laminas, the aberrometry parameters improved in almost all the study cases.

Visual acuity enhancement has been slightly better with the implant of negative meniscus lenticules than with the implant of planar laminas (14, 29, 31, 33), but the goal of the stromal implant should be to improve visual acuity and at the same time significantly increase the corneal thickness in patients with advanced keratoconus. Future studies are needed to improve the results in all these studies cited above.

Currently, a multicenter **SLAK** research group is working on the generation of customized lenticules that could combine the advantages of both planar (higher thickness increase and availability for eccentric cones) and negative meniscus-shaped lenticules (higher flattening effect).

The use of recombinant human collagen, synthesized in yeast and chemically cross-linked demonstrated to be feasible (35, 36), improved the visual acuity, nevertheless, a large variation of corneal thickness was obtained among the cases of this study up to 24 months of follow-up, and high presence of fibrosis was observed.

The use of acellular laminas (10, 11, 13, 14), in which the lack of cells makes the experience out from being considered as a corneal graft, and by using autologous **MSCs** from a given patient, it was possible to transform allogenic grafts into functional autologous grafts, thus avoiding any risk of rejection. So far, the long-term follow-up did not show any complications with all the cases (14).

Other recent studies of endokeratophakia using regular **SMILE** lenticules have shown to be a feasible option to correct ametropias such as hyperopia, presbyopia or to increase the thickness in the keratoconic cornea (40). Nevertheless, the use of customized positive meniscus lenticules in keratoconus corneas is debatable (29, 39), as theoretically could worsen myopia usually encountered in such patients, and so planar or negative meniscus lenticules are always preferable for such purpose. Implantation of autologous fresh lenticules from **SMILE** for hyperopia was well-integrated into the host stroma (41, 42). The fact that refractive lenticules can be stored, and be later re-implanted, opens up the possibility of using lenticules for other purposes to restore ectatic corneas or providing an opportunity for reversing refractive surgeries (40).

All the conclusions of these described studies should be carefully considered since they are vulnerable to bias because all the studies included a small sample of patients. However, all the surgeries reported in this review showed initially to be potentially effective for the treatment of advanced keratoconus. In none of the reviewed studies, there was obtain any rejection or inflammation in the treated corneas. Further studies will be necessary to increase the number of cases, and to increase the evidence of the effectiveness of these corneal surgeries with keratoconus or with other corneal stromal dystrophies.

## Conclusion

Cellular therapy with implantation of autologous **ADASCs**, decellularized human corneal stroma, allogenic **SMILE** Lenticule, Corneal Inlay, and to a lesser extent recombinant Cross-linked Collagen were discussed in this review. As this procedure indeed reported refractive and corneal topography complications it also showed initially to be potentially effective for the treatment of advanced keratoconus. Such promising findings open a new perspective of therapy of the corneal stroma based on corneal stromal enhancement and regeneration. Future studies will expand the potential of the application of these new therapies in the treatment of corneal stromal diseases.

## Author Contributions

JLA: principal investigator, principal surgeon, study concept and design, analysis and interpretation of data, writing and critical revision of the manuscript, statistical revision, and supervision. MEZ: co-principal investigator, clinical and monitor director of the study, study concept and design, data collecting, analysis and interpretation of data, writing and critical revision of the manuscript, statistical revision, and supervision. MPdM: biological processing, corresponding data editing, and critical correction of the manuscript. JLAdB: second surgeon, corresponding data editing, and critical correction of the manuscript. KAJ: analysis, interpretation, editing of the data, and writing the manuscript. NM: stem cells therapy processing. All authors contributed to the article and approved the submitted version.

## Conflict of Interest

The authors declare that the research was conducted in the absence of any commercial or financial relationships that could be construed as a potential conflict of interest.

## Abbreviations

ADASCs: Adipose-derived adult stem cells
SMILE: Small incision lenticule extraction
PKP: Penetrating keratoplasty
SFII: Femtosecond laser-assisted intracorneal concave lenticule implantation
SILK: Small incision sutureless intrasotromal lamellar keratoplasty
UDVA: Unaided distance visual acuity
CDVA: Corrected distance visual acuity
MSCs: Mesenchymal stem cells
ECM: Extracellular matrix
h-ADASCs: Human Adipose-Derived Adult Stem Cells
PBS: Phosphate-buffered saline
IFS: IntraLase femtosecond laser
AS-OCT: Anterior segment optical coherence tomography
CLDVA: Contact lens visual acuity
CCT: Central corneal thickness
Thinnest point: Scheimpflug corneal topography Thinnest point
CV: Cornea Volume
Rx Sphr: Refractive sphere
Rx Cyl: Refractive cylinder
3rd order RMS: Third-order aberration RMS
4th order RMS: Fourth-order aberration RMS
HOA RMS: High order aberration RMS
LOA RMS: Low order aberration RMS
Anterior Km: Anterior mean keratometry
Posterior Km: Posterior mean keratometry
Kmax: Maximum keratometry
Topo Cyl: Topographic cylinder
FILI: Femtosecond intrastromal lenticular implantation
UV: Ultra-violet
CXL: Cross-linking
SLAK: Stromal lenticule addition keratoplasty
IOP: Intraocular pressure
DALK: Deep anterior lamellar keratoplasty
RHC: Recombinant human collagen
EDC: 1-ethyl-3-(3-dimethylaminopropyl) carbodiimide
NHS: N-hydroxysuccinimide
ALK: Anterior lamellar keratoplasty.
